# Concerns of AI use in evidence synthesis based practices: collective views from the community

**DOI:** 10.1186/s12874-026-02844-x

**Published:** 2026-03-28

**Authors:** Hannah O’Keefe, Claire Eastaugh, Feyza Yarar, Jen Taylor, Chris Marshall, Fiona Campbell

**Affiliations:** 1 NIHR Innovation Observatory, Newcastle University, Newcastle-upon-Tyne, United Kingdom; 2 Population Health Sciences Institute, Faculty of Medical Sciences, Newcastle University, Newcastle-upon-Tyne, United Kingdom

**Keywords:** Artificial Intelligence, Evidence synthesis, Systematic Reviews, Horizon Scanning, Concerns

## Abstract

**Background:**

The use of artificial intelligence (AI) in research has become one of the most hotly debated topics. This is particularly true for the field of evidence synthesis where automation through AI may lead to substantial time and resource savings. Many researchers see the potential benefits of using AI technologies, yet there is hesitation around embedding AI in practice. We explored the concerns of those working in the field of evidence synthesis through a series of online and in-person events.

**Methods:**

Data collection was conducted across two in-person and 2 online events: the Evidence Synthesis Hackathon (ESH) 2024, the Community, Opportunities, Research and Experience Information Retrieval (CORE) Forum, a Systematic Review Conversations (SRC) online seminar, and an online Horizon Scanning (HS) Survey. Inductive and deductive coding was utilised to synthesis data into broad themes and subthemes, independently for each event. A vote counting and ranking approach was used to triangulate data across events to capture convergent and divergent themes between participant groups.

**Results:**

Across the four events we acquired a total of 248 data points (from 80 respondents) and responses were broadly similar across cohorts. Through synthesis and triangulation, we identified 10 overarching themes. The most prominent themes were knowledge and skills, and data management, respectively. Skills loss, skills gap and job loss were highlighted within the knowledge and skills theme. Bias, confidentiality and reliability were prominent for data management. Lower ranking concerns included environment, economics, AI market and costs.

**Conclusions:**

These are valid apprehensions faced by researchers across the field of evidence synthesis and should be considered in the broader discussion of AI. Development of rigorous methodologies and guidance may help to overcome these issues by facilitating responsible and transparent use of AI.

**Supplementary Information:**

The online version contains supplementary material available at 10.1186/s12874-026-02844-x.

## Background

In recent years, we have seen a data overload, with data sizes doubling every two years. The health and social care industry generates approximately 2% of the worlds data [1]. This includes information generated through health services, pharmacology companies and medical technologies. Whilst health and social care data comes in many formats, it is clear that the internet is now the most widely used source of information with search engines such as Google and Bing being the cornerstones of internet browsing [2]. Data in a digital format reached 33 zettabytes in 2018, with approximately 30% of this digital content being health and social care related [3]. This complex data landscape is being seen across disciplines, not just health and social care, meaning that automation methodologies are highly sought after to enable researchers to conduct robust and timely syntheses of information.

Artificial Intelligence (AI) is a rapidly growing area with wide applicability to evidence synthesis methodologies. Whilst embedding automation methodologies in evidence synthesis is not a new concept, significant attention has been garnered since the launch of Generative AI (GenAI) methodologies, specifically the release of ChatGPT in 2022 [4]. Techniques such as entity recognition and relationship extraction are well documented and validated in evidence synthesis but rarely used in practice. Researchers are excited about the advancement of AI, and this brings a world of promise for new automation methodologies [5]. Yet, concerns remain about the use of any form of AI, resulting in hesitancy in progressing in the field. This is partly due to the relevance and influence that evidence syntheses hold in decision-making for policy and practice. Over recent years, redundant or misleading systematic reviews have been abundant [6]. This type of mass production can be expedited by the use of AI methodologies [7]. We are seeing many researchers, research funders and institutions releasing statements about the responsible use of AI in research in an attempt to combat mass production and a growing body of misinformation [8]. Similar efforts are being brought forward by policymakers and governing bodies, such as the National Institute of Health and Care Excellence position statement on the use of AI in evidence generation [9]. Given the importance of ensuring evidence syntheses are accurate and trustworthy, we must address the concerns of researchers to better facilitate the use of AI methodologies.

Here, we exploited recent events and surveys to call on the collective intelligence of the evidence synthesis community. We wanted to know what concerns those embedded in the field have around the use of AI in evidence synthesis. Thus, we carried out a mixed-methods study using qualitative data collection and quantitative synthesis tasks to elicit expert opinion through the Evidence Synthesis Hackathon (ESH), the Community, Opportunities, Research and Experience Information Retrieval (CORE) Forum, a Systematic Review Conversations (SRC) seminar, and an online Horizon Scanning (HS) Survey.

## Events

The annual ESH aims to bring evidence synthesis experts and technical developers together to tackle automation problems in the field. It does this through open collaborative working, focussing on building or advancing an ecosystem of open-source tools to aid evidence synthesis through collective development, testing, and promotion [10]. The ESH also focusses on building networks and developing capacity to deliver automation methodologies. It supports 65 global institutions and has facilitated the initiation of over 30 projects, enabling 20 + tools to be made available to the community since 2018. To date, over £25,000 GBP has been raised through donations to support the ESH and its conference counterpart ESMARConf. These generous donations have allowed the initiative to continue to deliver events for free and support lower income individuals to attend. The ESH 2024 was well attended and led to collaborative efforts to progress 10 projects, including Bibfix [11], Citationchaser [12], and stopping criteria for machine learning algorithms.

The inaugural CORE Forum was held in January 2025 in-person at Newcastle University [13]. It was funded by the National Institute of Health and Care Research (NIHR) Innovation Observatory and NIHR Methodology Incubator. The NIHR is a leading funder for public health and social care research in the UK, spending over £458 million GBP in research funding for 2023/24 and a further £143 million GBP on training and career development, as well as significant investments in infrastructure, data and digital, and official development assistance [14]. NIHR is closely aligned with the National Health Service (NHS) through the National Institute of Health and Care Excellence (NICE) and the Department of Health and Social Care (DHSC), leading research through policy and into practice. The NIHR Methodology incubator, established in 2020, supports methodologists working in applied health and care research, including those from the NIHR Innovation Observatory which is home to one of the largest groups of information professionals in the UK [13, 15].

SRC is a series of free online seminars hosted by the library service at Lancaster University [16]. It has been running since 2023 with the aim of providing informative talks on systematic review methodologies. Speakers are invited to share their research and experiences with an engaged audience of peers, students and anyone with an interest in evidence synthesis. Attendance typically ranges from 120 to 180 people but can accommodate up to 300, and the recordings are made available on the library YouTube channel [17].

The HS Survey was a one-off survey designed and distributed via online platforms by the NIHR Innovation Observatory [13]. With over 80 employees, the NIHR Innovation Observatory has substantial networks with national and international organisations [13]. As well as housing one of the largest known groups of information specialists, the NIHR Innovation Observatory hosts a substantial team of data science and AI specialists, evidence synthesists, horizon scanning analysts, public health researchers, and professional services specialists.

## Methods

The four event were chosen as they represented a broad range of professions within the evidence synthesis community and were a pragmatic choice in terms of accessibility. A mixed-methods approach was taken to collate data, utilising a mix of collaborative working styles and questions as detailed below (Table 1).

**Table 1:** Questions posed to each cohort at the events

**ESH**
*‘Please bullet point any concerns you have about using AI in evidence synthesis. There are no right or wrong answers*,* and nothing is too big or too small. These bullets may be used as* *anonymised* *quotes in the final manuscript. Please be as open and honest as possible.’*
*‘We are also interested in whether you have heard concerns about using AI in evidence synthesis during conversations with colleagues or through your networks. Even if you do not agree with the concerns*,* we would like you to bullet point these below. This will help us gather a broader picture and generate themes.’*
**CORE Forum**
*‘How do you use AI?’*
*‘How do you want to use AI?’*
*‘What concerns do you have?’*
*‘How do we spot if any information we review/critique has been AI generated?’*
**SR Conversations**
*‘What concerns you about using AI in your research?’*
*‘What would a new AI based methodology look like to you?’*
**HS Survey**
*‘How often do you come across horizon scanning in your own work?’*
*‘How are you involved in horizon scanning? If “Other” Please tell us how you are involved in horizon scanning.*
*‘Do you use any form of artificial intelligence in part of your daily work? For this question*,* we would like to know about your work in general*,* not just horizon scanning.’*
*‘What type of artificial intelligence do you use?’*
*‘Please tell us what you use artificial intelligence for’*
*‘Do you use any specific named tools?’*
*‘In general*,* how confident are you at using artificial intelligence methods for small tasks? By small tasks we mean things like document editing*,* generating images or visuals*,* transcribing or summarising meeting recordings’*
*‘In general*,* how confident are you at using artificial intelligence methods for advanced tasks? By advanced tasks we mean things like classification systems*,* data handling and extraction*,* and building your own models’*
*‘Thinking about these two questions*,* can you explain why you feel this way?’*
*‘Do you think artificial intelligence should be used to support horizon scanning?’*
*‘Can you expand on why you think this?’*
*‘If artificial intelligence was to be used*,* which parts of the horizon scanning process do you think would benefit most and why?’*
*‘If artificial intelligence was to be used*,* which parts if the horizon scanning process do you think are least likely to benefit and why?’*
*‘Would you feel comfortable relying on artificial intelligence driven recommendations or outputs?’*
*‘In general*,* how comfortable/uncomfortable would you feel using artificial intelligence driven recommendations or outputs?’*
*‘Thinking of these two questions*,* can you expand on why you feel this way?’*
*‘Do you think data quality and reliability are positively or negatively impacted by using artificial intelligence methods?’*
*‘Can you expand on why you think this?’*
*‘Are there specific factors associated with artificial intelligence that you think could positively or negatively affect data quality and reliability?’*
*‘Thinking about conversations you may have had about using artificial intelligence in a horizon scanning context*,* have other people mentioned things that worry or concern them?’*
*‘Can you expand on what concerns or worries people have mentioned? Please be as detailed as possible’*
*‘What are the top 5 things that worry or concern you about using artificial intelligence to support horizon scanning?* *It may be useful to think about*: * • Implementation factors - such as transparency*,* accuracy*,* bias*,* explainability*,* ethics*,* cost* * • Personal factors - such as knowledge*,* understanding*,* training needs*,* time commitments* * • Global factors - such as environment*,* workforce*,* labour*,* economics’*
*‘Do you have any other comments you would like to make?’*

### Data collection

#### ESH

We asked delegates at the Evidence Synthesis Hackathon November 2024 to engage with a collaborative data collection exercise using Google Docs. As part of this, we asked them to document their personal concerns about using AI in evidence synthesis. We also asked delegates to document any concerns they have heard mentioned during conversation with colleagues or within their networks (Table 1). Data was collected in two waves on the first and second day of the event, respectively. Ethics approval was sought from Newcastle university [Ref: 52906/2023].

#### CORE forum

Details of the CORE forum AI discussions have been published elsewhere [18]. Briefly, a one-hour knowledge cafe was held during the CORE Forum where participants had the option to move between six discussion tables: AI; search sources; experience; networking; professional development; and keeping the momentum of the forum going. Four questions were given to initiate conversation around AI (Table 1). No limitations were placed on the type of AI or use case scenarios. QR codes leading to an online collaborative space (Padlet) [19] and poster boards for writing were available throughout the day. The CORE forum was deemed exempt from ethical approval processes as comments were collected anonymously for non-research purposes. However, delegates were made aware that their contributions may be used retrospectively for research purposes.

#### SR conversations

Delegates at the SR Conversations session May 2025 were asked to participate in an online data collection exercise using online collaborative space (Padlet) [19]. Two questions were posed (Table 1) and participants were asked to leave anonymous contributions. Those surrounding ‘concerns of use’ were taken forward for analysis. Ethics approval was sought from Newcastle university [Ref: 59164/2023].

#### HS survey

Qualtrics was used to design a survey with a mix of single select, multi select, and open-ended questions (Table 1, Supplement 1) [20]. Questions were initially developed by HOK and reviewed by CM. The survey was distributed via a communications plan developed by JT, consisting of social media threads, newsletters and email lists. The survey remained open for contributions across a three-month span (April 2025 - June 2025). Ethics approval was sought from Newcastle university [Ref: 57600/2023].

### Analysis

Each qualitative response was used as a single data point. Data points were synthesised into broad themes and subthemes using inductive and deductive coding, following the thematic coding methodology proposed by Braun and Clarke [21, 22]. At the ESH, day-1 data was synthesised using an inductive approach where FY derived themes from the data, and themes were checked by HOK. On day-2, a deductive approach was taken to incorporate additional responses into the day-1 themes. Inductive approaches were used by HOK and CE for the CORE Forum, and by HOK for the SR Conversations and the HS Survey. Quantitative analysis was used where appropriate for the HS Survey responses. HOK was responsible for the coding across all events to minimise inconsistencies and ensure harmonisation of codes. Subthemes created during these independent analyses were triangulated to identify convergent and divergent themes across the participant groups by HOK and critically reviewed by CE. Furthermore, a vote counting approach was used to quantify and rank the themes and subthemes. This quantification was used to identify the most frequently counted concerns amongst the cohorts. However, participants may have attended more than one event, and the findings could be overinflated if the same response was given at multiple events. We have attempted to minimise this risk by presenting the findings both individually for each event (Supplement 2) and as a whole (see collective themes and data triangulation section). Finally, we did not consider the ‘depth’ of each concern; thus, no conclusions of importance or weighting can be drawn from our findings.

## Results

### Participant characteristics

#### ESH

The ESH 2024 had 32 registered delegates in attendance. Of these, nine are aligned with data scientists or development, 18 are aligned with evidence synthesis, one information professional, one allied health professional and one statistician. The global spread of participants was broad: India, Norway, Germany, Portugal, USA, Canada, and the UK; most participants were situated in the UK (*n* = 24, 75%). Thirteen participants (40%) contributed to the data collection.

#### CORE forum

A total of 131 delegates, from 10 countries and nine different types of organisations, where present at the CORE Forum. As with ESH, the global spread of participants was broad: USA; Slovakia; Brazil; Canada; Germany; Morocco; Netherland; Nigeria; Sweden; with most being situated in the UK (*n* = 119, 91%). Twenty-one participants (16%) contributed to the data collection.

#### SR conversations

The event had over 400 registered delegates, however the actual attendance rate fell to approximately 170. Further information regarding participant characteristics were unavailable. Informally, the delegates were thought to be a mix of evidence synthesis specialists, information professionals and students studying systematic review methodologies. Thirty-four participants (20%) contributed to data collection.

#### HS survey

Seventeen responses were received from the survey, two of which were ineligible for participation and did not proceed beyond the eligibility questions. Two were accepted but failed to fill in the remaining questions, and one completed 11 of the 23 questions. Incomplete survey responses were removed from the analysis. Of the 12 participants (71%) that completed the survey, 10 actively conduct horizon scanning, one utilises the outputs from horizon scans within their own work, and one comes across horizon scanning in their work but is not actively involved or using the outputs. Participants were located in Newcastle, Durham, Teesside, York, and London.

### Synthesis of themes and subthemes

**Fig. 1:**
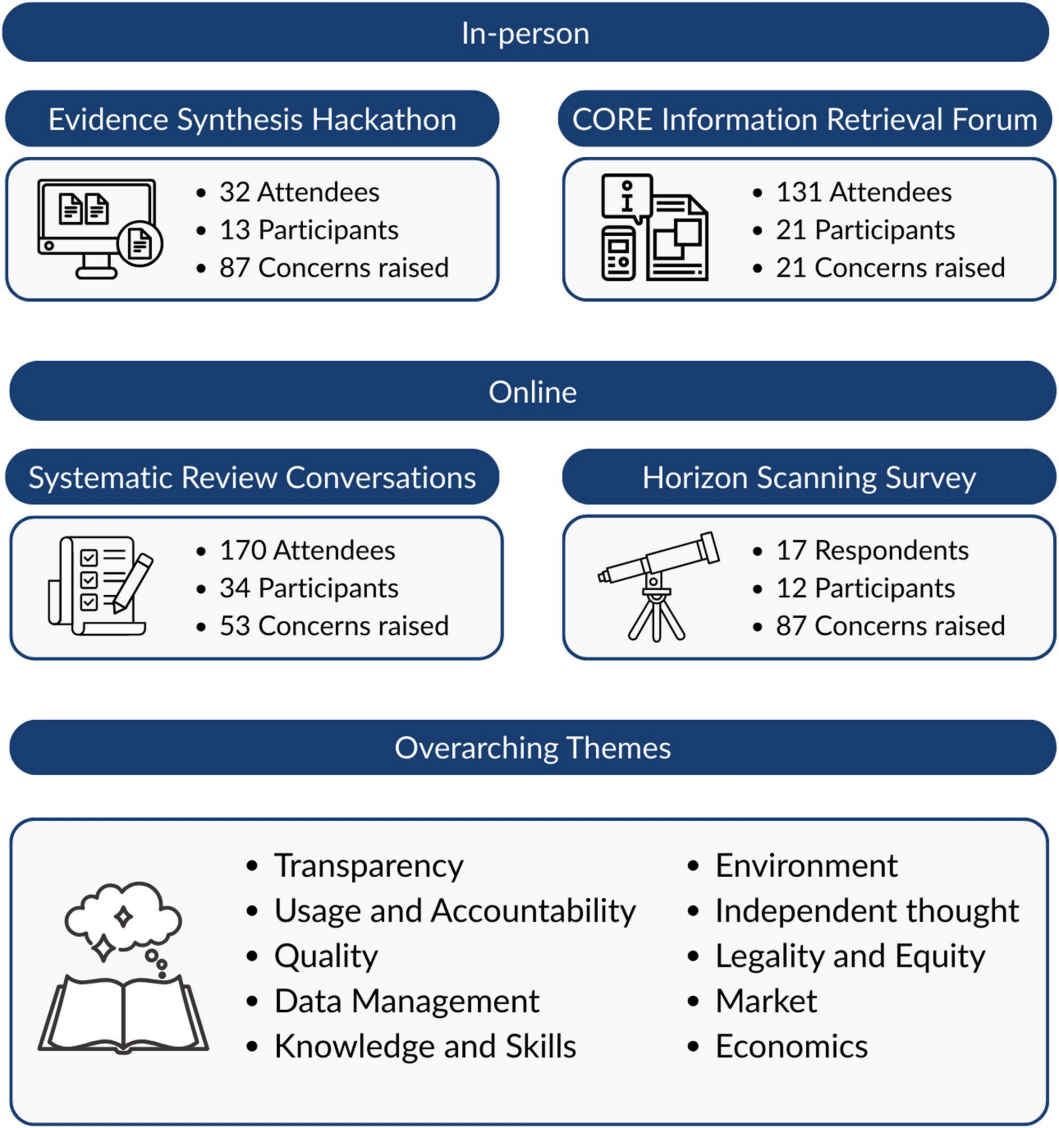
Depiction of the in-person and online events, including number of delegates at the event, number of participants contributing to this study, and number of data points (‘concerns’) raised by each cohort, and the overarching themes from the data synthesis and triangulation

A total of 248 data points (from 80 respondents) were identified across the events (Fig. 1).

We provide a brief overview of the findings below; a full description of the individual event results can be found in supplement 2.

#### ESH

We gathered 70 data points for first-person accounts. These were thematically grouped into seven broad themes: errors, outcomes and functionality (*n* = 26); the workforce (*n* = 14); consensus, transparency and reporting (*n* = 10); tools, methodologies and processes (*n* = 9); human rights and politics (*n* = 6); environment (*n* = 4); and economics (*n* = 1).

For third-person accounts, we collated 17 data points across five broad themes: human rights and politics (*n* = 7); workforce (*n* = 4); environment (*n* = 3); errors, outcomes and functionality (*n* = 2); and consensus, transparency and reporting (*n* = 1).

#### Core forum

The AI table at the knowledge café was attended by up to 16 participants at any one time. The AI discussion platforms (knowledge café, online space and boards) received a total of 49 contributions throughout the day. We identified four broad themes arising from the discussion on AI: critical perception (*n* = 21); current uses (*n* = 19); training wants/needs (*n* = 7); and specific tools (*n* = 2). Detailed findings of the CORE Forum AI discussion and independent synthesis have been published elsewhere [18]. Here, we concentrate on the critical perception findings, and the 28 data points arising under other themes are not considered in our total count.

#### SR conversations

We received 53 contributions to the question regarding concerns. We also received a further 18 comments regarding what a new methodology should look like, however these were not taken forward for analysis in this space.

We derived four broad themes from across the 53 responses: functionality and reporting (*n* = 27); workforce (*n* = 17); ethics (*n* = 5); and environmental (*n* = 4). Within functionality and reporting, almost half of the responses related to transparency and reproducibility (*n* = 13).

#### HS survey

We utilised 41 free text answers given across the survey to derive themes, resulting in five broad themes: Confidence (*n* = 5); Readiness levels (*n* = 9); Data management (*n* = 14); Human factors (*n* = 11); and Environment (*n* = 2). We specifically asked participants for the ‘top 5 concerns’ they had about using AI to support horizon scanning and received 46 responses. These were broadly categorised, with reliability/accuracy and training, skillset and guidance being identified as the main concerns (Table 2). This echoed the thematic findings where data management and human factors were prominent.

**Table 2:** Responses from HS survey participants when asked to list their top 5 concerns

Concern	Number of Responses
Reliability/Accuracy	12
Training, skillset and guidance	9
Environment	4
Ethics	4
Transparency	4
Workforce/labour	4
Confidentiality and data protection	2
Cost	2
Job security	2
Enforced use	1
Equity, Diversity, Inclusion	1
Reproducibility	1

When asked to select how they felt about using AI driven recommendations or outputs, half of the participants were somewhat uncomfortable or extremely uncomfortable (*n* = 6). A quarter were neutral (*n* = 3) and the remaining where somewhat comfortable (*n* = 3). Interestingly, 10 participants indicated that they thought AI could be used to benefit horizon scanning, yet only three thought data quality and reliability would be positively impacted by using AI.

### Collective themes and data triangulation

We looked across the subthemes of each set of responses and the top concerns isolated from the HS survey to identify convergent and divergent themes (Table 3). Ten overarching themes were present (Fig. 1; Table 3), with at least two of the events contributing to each theme. Whilst we outline the vote counting here, the potential for double counting or overinflation must be kept in mind when interpreting the results.

**Table 3:** Data triangulation across cohorts, with subthemes generated by individual thematic syntheses clustered into 10 overarching themes

Overarching theme	ESH subthemes (*n* = 87)	CORE subthemes (*n* = 21)	SRC subthemes (*n* = 53)	HS subthemes (*n* = 41)	HS top concerns (*n* = 46)
*Knowledge and skills* (*n* = 60; 24%)	Robustness (*n* = 3)	AI promotes taking shortcuts (*n* = 3)	Skills loss (*n* = 3)		
	Skills gap (*n* = 9)	Lack of understanding or expertise, avoidance (*n* = 3)	Skills gap (*n* = 10)	Lack of skills, knowledge, confidence (*n* = 6)	Training, skillset and guidance (*n* = 9)
	Rapid advances (*n* = 3)			Human understanding (*n* = 3)	
		Appropriate stopping points (screening related) (*n* = 1)	Ignorance (*n* = 2)		
	Job security (*n* = 2)		Job losses (*n* = 1)		Job security (*n* = 2)
*Data management* (*n* = 46; 18%)	Bias (*n* = 3)	Inherent bias of ingested data (*n* = 1)	Bias (*n* = 5)		
				Confidentiality and data protection (*n* = 2)	Confidentiality and data protection (*n* = 2)
	Plagiarism and duplication (*n* = 2)				
	Feedback loops (*n* = 3)			Data quality and verification (*n* = 3)	
	Hallucination (*n* = 4)			Errors and misinformation (*n* = 9)	Reliability and accuracy (*n* = 12)
*Transparency* (*n* = 40; 16%)	Lack of transparency (*n* = 7)	Trustworthiness transparency, and reproducibility of data (*n* = 4)	Transparency and reproducibility (*n* = 13)	Lack of confidence and trust in outputs (*n* = 5)	Transparency (*n* = 4)
	Lack of reporting standards (*n* = 2)	Insufficient guidance or standards (*n* = 3)	Lack of reporting standards (*n* = 1)	Thorough reporting (*n* = 1)	
*Legality and equity* (*n* = 31; 12%)	Invisible labour (*n* = 3)		Legality and morality (*n* = 6)		Workforce and labour (*n* = 4)
	Inequity (*n* = 8)		Equity (*n* = 1)		Equity, diversity, inclusion (*n* = 1)
	Consequences for humanity (*n* = 3)				Ethics (*n* = 4)
	Uncertainty (*n* = 1)				
*Quality* (*n* = 27; 11%)	Quality (*n* = 17)	Ai tools are not yet fit-for-purpose (*n* = 4)	Quality (*n* = 3)	Not fit for purpose (*n* = 1)	
	Reproducibility (*n* = 1)				Reproducibility (*n* = 1)
*Environment* (*n* = 18; 7%)	Environmental impact (*n* =3)	Environmental impact (*n* = 1)	Environmental impact (*n* = 4)	Environmental impact (*n* = 2)	Environment (*n* = 4)
	Carbon impact (*n* =1)				
	Energy usage (*n* = 3)				
*Independent thought* (*n* = 11; 4%)	Lacking independent thought (*n* = 1)	Lack of critical thinking and critical appraisal (*n* = 1)	Critical appraisal (*n* = 1)	Supporting role (*n* = 8)	
*Usage and accountability* (*n* = 7; 3%)	Proportionate use (*n* =1)				Enforced use (*n* = 1)
	No consensus of use (*n* = 3)				
			Accountability (*n* = 1)	Ownership (*n* = 1)	
*Market* (*n* = 4; 2%)	Tool saturation (*n* = 1)		Market dominance (*n* = 2)		
	Reduced innovation (*n* = 1)				
*Economics* (*n* = 4; 2%)	Economics (*n* = 2)				Cost (*n* = 2)

Knowledge and skills was the most prominent overarching theme, with contributions from each independent cohort, accounting for 24% of the total responses. A lack of skills or understanding (i.e. skills gap) was by far the highest reported idea, demonstrating broad convergence across cohorts.

*HS: “I want to use AI in my everyday for more than just editing purposes*,* I see how it can be used but*,* as has been noted*,* I lack confidence in AI and knowledge in its effective use.”*

*ESH: “Increasing skills gap between data scientists and more ‘traditional’ ES reviewers – are those of us who aren’t especially skilled in data science going to be left behind?”*

Data management was also widely reported, accounting for 18% of the total responses. Ideas around errors in outputs and the reliability of results accounted for a large proportion of this theme. However, it was the HS survey cohort who raised this issue the most. It was also the most highly scoring in the top 5 concerns given by this group. Interestingly, the introduction of bias or inherent biases of AI were not raised by this group but were raised by the three systematic review-based cohorts. Similarly, confidentiality and data protection were only raised by the HS survey group. Transparency accounted for a further 16% of the total responses. Two core impressions where projected within this theme: lack of transparency and lack of reporting standards. These two ideas where wholly convergent across the cohorts. Many individuals expressed their discomfort and described challenges with a lack of thorough reporting:

*HS: “It would depend on whether I knew which parts of the process had used AI*,* in what way*,* and whether it was well reported. If it was used for screening I’d be very comfortable. If they said ‘we used AI’ with no more detail I’d be uncomfortable. If I thought there was no human in the loop I’d be extremely uncomfortable.”*

*CORE: “Transparency in use of AI - don’t know where or how it is being applied. Standards for describing this are limited*,* not sure how to enforce. Challenge for editors. (Something we talk about as editors.)”*

*ESH: “The use of AI (if any) needs to be incorporated into the PRISMA checklist*,* so researchers are forced to record whether/how they have used it.”*

Legality and equity were reported by the ESH and SRC cohort. Whilst the HS cohort did not express ideas around this, they did rank ethical issues within the top 5 concerns. Together this totalled 12% of the total responses. The information cohort diverged from this and did not report any legal or equity concerns.

Quality of AI was reported by all cohorts, giving 11% of the total responses. It was widely suggested that AI is not fit for purpose and/or results in poor quality work.

*HS: “In my experience*,* AI as of yet is unable to effectively perform complex tasks like data extraction as it cannot differentiate between contextual figures (e.g.*,* ‘No technologies in this area have ever achieved over 80% specificity’*,* it will extract ‘achieved over 80% specificity’) and this leads to inaccurate data that needs to be redone by a human in the end.”*

Although environmental impact was raised across all cohorts, it bared little emphasis comparatively with only 7% of the total responses. All cohorts cited environmental concerns, but only the ESH cohort delineated these concerns into carbon impact and energy usage.

Independent thought was raised by all cohorts but only accounted for 4% of the total responses. It was most pronounced in HS survey cohort with 8/11 comments coming from this group. Yet, it was not mentioned in top 5 concerns of any of the survey participants.

Usage and accountability were not a common theme, with only 3% of the total responses relating to this. Nearly half of the comments were around consensus of use which all came from the ESH cohort. For example:

*ESH: “No consensus on how good AI has to be (at screening, data extraction etc)”*

Economics and the current AI market were less widely reported, with only two of the cohort raising these points and each theme accounting for 2% of the total responses. Costs were not mentioned within the HS survey responses but were listed as a top 5 concern by two respondents.

## Discussion

The world of AI is increasingly prevalent as it becomes embedded in our everyday lives; from the way we communicate to how we monitor our health and wellbeing it is almost inescapable in modern society. This has filtered through to the evidence synthesis research community, with the conundrum of exponentially expanding volumes of data and pressures to synthesise information at an increasingly rapid rate [23, 24]. AI methodologies have been poised as the answer to this dilemma for quite some time, yet the use of established, validated tools are not routine and novel methodologies are cautioned [25, 26]. We called upon evidence synthesis and automation experts to identify core concerns with the use of AI in evidence synthesis projects. By triangulating the data across events, we have started to build a picture of common perceptions across the cohorts (Fig. 1).

Amongst the comments was a plethora of ideas that are well-known in research circles. The lack of reporting standards and consensus on how AI should be used ran alongside comments regarding the ‘black box’ of AI and its propensity for hallucination [27]. Those working in evidence synthesis are used to adhering to rigorous processes and reporting, with a wealth of guidance and standards published by leading organisations such as Cochrane and JBI. Researchers, funders and governing bodies are working towards consensus by producing guidelines for responsible use of AI [8, 9]. Substantial investment of time and money are being diverted to support this. In time, we will see robust guidelines and reporting checklists, similar to those we currently have to support evidence synthesis from organisations such as PRISMA and Cochrane [28, 29]. To date, two sets of guidance called RAISE have been produced: Responsible Generative AI for small to medium enterprises (SMEs) in the UK and Africa: RAISE guidelines [30], and RAISE: Responsible AI use in evidence SynthEsis guidelines [8]. Whilst these both aim to give structure and regulation to AI use, the naming may lead to some confusion about which guidelines to follow, particularly in the horizon scanning space where researchers commonly engage with SMEs and for those building their own AI tools [13]. Nevertheless, such guidance has been called for, as seen here in the responses, and may help to improve individuals confidence in using AI responsibly. We also see a lack of confidence in using AI and a perceived skills gap in our responses. Perhaps, the lack of structured guidance and a wariness of not fully comprehending the models is driving this. No single theme or cohort had wildly divergent responses. However, the CORE cohort raised fewer concerns about skills gap than other cohorts. As demonstrated in the findings of the CORE Forum, information professionals are not averse to using AI and potentially see the skills gap as an opportunity to learn and develop rather than a concern [18].

The issues raised around data management suggest a pattern of divergence between those working in systematic reviews (ESH, CORE, SR cohorts) compared to those in horizon scanning (HS cohort). This theme may be related to the methodologies used by each cohort. Systematic reviewers routinely perform bias assessments and will be well aware of the potential for bias, whereas risk of bias assessment is less common in horizon scanning. Conversely, horizon scanning is much more likely to use some form of confidential data, whereas systematic reviews are based on published literature. Interestingly, accounts of errors, misinformation and reliability of outputs were raised considerably more often by the HS cohort. Perhaps this is related to the immediate nature of horizon scanning where outputs are often given directly to decision makers as opposed to going through a lengthy but extensive peer-review and publication process. Further research is needed to identify if these are indeed true patterns of divergence, and if so, what the underlying reasons for this may be.

Issues around ownership and where the accountability lies were raised. Although these comments were limited, they are important and warrant further discussion amongst the community. If AI is to be used in evidence syntheses that inform policy and practice, such as health technology appraisals, who is accountable if misinformation is introduced. There is the potential for a legal component to this, particularly in relation to medical malpractice. This should be explored with medico-legal practitioners and governing bodies to understand the full extent of these implications. Furthermore, there is an inherent danger that AI’s big “hype” will result in researchers using AI for tasks where there are no gains compared to traditional methodologies. Disproportionate or enforced use is not without consequences, considering aspects such as the high energy demands associated with the use of some of these methodologies. Reports have emerged evidencing that leading AI corporations have increased carbon emissions by up to 50% since GenAI methodologies reached public consciousness [31]. There are estimates that generative AI uses approximately a third more energy than other software to complete a task [32]. Energy consumption was recurrently cited as a concern under the theme of environment. Comparative energy consumption analyses for evidence synthesis with and without AI are warranted.

Whilst concerns about the environmental impact of AI were raised across all cohorts, this was to a lesser extent than concerns about the impact on fellow human beings. Particularly the implications of AI for the exploitation of workers and biases for minority groups. This is known issue which has started to attract research funding to explore the impact and scale of such ‘invisible’ workforces [33]. The picture of ecological consequences and manipulations of human rights poses an ethical and moral conundrum for researchers who are under considerable pressure to perform faster, more rapid evidence syntheses. On the one hand, rapid evidence collection allows policy and decision-makers to introduce new legislation and practices for the benefit of humankind at an advanced pace. On the other, these detriments to our environment and fellow humans can be seen as inhumane and may lead to more catastrophic outcomes in the long term, undoing any good achieved by using AI in the first place. Here, these ‘invisible’ workforce issues were raised in respect to the North-South divide. However, the participants at these events where mostly from the United Kingdom, with some international delegates but little representation of low-income and middle-income countries. The CORE Forum had the most diverse delegates in relation to country yet raised no concerns about humanity. We did not explore the nationality of delegates at any of the events, nor which countries they may have worked in previously, and we cannot build any inferences based on country alone. Further research is warranted to understand the global perceptions of AI use and how these may differ by global divides and priorities.

Interestingly, those raising issues around human impact also considered the current market and economic viability of AI tools. Again, this was seen in relation to the North-South divide. There is huge potential for the evidence synthesis community to bring about change to research practices, equity and the open science movement [34]. Developing the infrastructure to train or fine tune methodologies which are not under control of private for-profit companies and are instead curated by and for evidence synthesis professionals, may reduce some of the human rights and economic concerns that have been raised here. A necessity to share raw data, code or workflows may indeed prove beneficial and lead to a fundamental change in research culture whereby open science becomes routine practice [34]. An open science approach may relieve worries about fabricated research generated by AI and lead to more transparency about the reliability of studies.

### Strengths and limitations

A wide range of specialities, including information professionals, evidence synthesis methodologists, computational experts, and horizon scanning professionals, were responsive to the activities. The participants all had some level of experience with systematic reviews and evidence synthesis, making them an ideal audience to answer our research question. This enabled us to gather a range of viewpoints and led to an enriched synthesis.

We must consider the implications for bias within this study. Given participants were aware that the information provided in this study may be submitted for publication, there is a possibility that participants could include information that they wish to see in print, either consciously or subconsciously. Using a multi-event approach allowed everyone to have a voice, and the anonymisation gave participants the opportunity to challenge ideas and statements without recourse. The combination of multiple data sources and perspectives has given depth and breadth to understanding the concerns researchers face, with many similar concerns being raised across the independent cohorts.

We must also consider that the conversation around using AI in evidence synthesis has been ongoing for more than 20 years. There were no outliers in the responses which could indicate that responses are engrained from the long-standing conversation rather than free thinking. Although difficult to assess if this is the case, it is a form of potential bias. Perhaps deeper research using psychosocial interventions such as critical perception exercises, nominal grouping or fostered debates may help to break any existing herd mentality.

Whilst using multiple data sources and triangulation has given strength to the findings, we used an inductive coding approach on individual datasets rather than a deductive framework across the entire dataset. This may have implications for consistency of coding. We minimised this by employing a single researcher consistently across all data sets, with support from second researchers across the in-person events. Furthermore, there is a possibility that participants contributed the same comment to more than one event resulting in double-counting. Thus, a single deductive analysis may have led to overinflation of the findings. We have provided individual analyses with the triangulated data to overcome this.

We found that both in-person and online events had good participation rates. However, when comparing the number of delegates to the number of participants and responses, events with lower numbers of delegates (ESH, HS Survey) had much higher participation rates. Delegates in large cohorts may have felt that their point had already been raised and therefore did not contribute. Extending this research to smaller focus groups or individual interviews may help to identify if data saturation has indeed been met.

## Conclusions

AI presents an opportunity for experienced interdisciplinary specialists to facilitate ways that improve and streamline how evidence syntheses are conducted. However, AI use should not come at the expense of the environment and widening inequalities. When used responsibly with a transparent approach underpinned by guidance, the use of AI may improve research accessibility. Before AI is used to facilitate increasingly complex tasks there must be robust processes, methods and guidance by which concerns such as the ones outlined in this study are addressed.

## Supplementary Information

Supplementary Material 1.

Supplementary Material 2.

## Data Availability

The datasets used and/or analysed during the current study are available from the corresponding author on reasonable request.

## Abbreviations

AI: Artificial intelligence
GenAI: Generative artificial intelligence
ESH: Evidence Synthesis Hackathon
CORE: Community, Opportunities, Research and Experience Information Retrieval forum
SRC: Systematic Review Conversations seminar
HS: Horizon Scanning survey
NIHR: National Institute of Health and Care Research
NHS: National Health Service
NICE: National Institute of Health and Care Excellence
DHSC: Department of Health and Social Care
