# Supplementary material for: Concerns of AI use in evidence synthesis based practices: collective views from the community
Source: BMC Med Res Methodol. 2026 Mar 28;26:106. doi: 10.1186/s12874-026-02844-x (PMC13151256; doi:10.1186/s12874-026-02844-x)
Supplement: Supplementary file 1 — Supplementary Material 1. [file 12874_2026_2844_MOESM1_ESM.docx]

# Supplement 1: Synthesis of themes and subthemes

A total of 246 data points (from 80 respondents) were identified across the events (Figure 1).

*Figure 1. Depiction of the in-person and online events, including number of attendees to the event, number of participants contributing to this study, and number of data points (‘concerns’) raised by each cohort, and the overarching themes from the data synthesis and triangulation.*

#### ESH

We gathered 70 data points for first-person accounts. These were thematically grouped into seven broad themes: errors, outcomes and functionality (n = 26); the workforce (n = 14); consensus, transparency and reporting (n = 10); tools, methodologies and processes (n = 9); human rights and politics (n = 6); environment (n = 4); and economics (n = 1).

The highest rate of first-person concerns fell under the error, outcomes, and functionality theme, with eight of those based around quality. Participants expressed concerns around human laziness and how this might affect the meaning and reliability of the synthesis:

*ESH: “People cut corners, I do not expect ‘continuous and very careful/alert evaluations’ to become the norm – they require too much effort, so systematic errors will creep in and potentially become pervasive”*

*ESH: “Evaluations are hard, measuring accuracy is difficult and cannot be done on a one-shot basis. If we’re dealing with review updates or living reviews, what was measured 6 months ago might not be true now”*

Comments around skills gaps were also common, with seven of the workforce concerns falling into this subtheme. Worries of being left behind as methodologies advance or losing the human connection with the data that leads to an innate understanding were articulated:

*ESH: “Increasing skills gap between data scientists and more ‘traditional’ ES reviewers – are those of us who aren’t especially skilled in data science going to be left behind?”*

*ESH: “Reduced intellectual engagement with the data, what it means, and how it can be translated and applied.  Even screening references – the one thing everyone wants automated – can be useful to engage with as a reviewer. It gives me a keen sense of what is in the field. I can’t even just rely on other reviewers’ data extraction without looking myself at the papers. Immersion in the data is key to reviewing.”*

A lack of transparency and no consensus of use were highlighted amongst comments regarding consensus, transparency, and reporting, with four and three of the themes data points, respectively. Mentions of ‘black-box’ were common, with an emphasis on the need to address this issue. Further points were raised about the challenges faced by editorial boards and others when there is no consensus on threshold requirements of AI or current requirements for reporting:

*ESH: “[as others have noted, but to emphasize again:] Transparency around how exactly the models work is not standard. There are many ‘black-box’ models and package”*

*ESH: “No consensus on how good AI has to be (at screening, data extraction etc)”*

*ESH: “The use of AI (if any) needs to be incorporated into the PRISMA checklist, so researchers are forced to record whether/how they have used it.”*

For third-person accounts, we collated 17 data points across five broad themes: human rights and politics (n = 7); workforce (n = 4); environment (n = 3); errors, outcomes and functionality (n = 2); and consensus, transparency and reporting (n = 1).

When considering third-person accounts, ideas around inequity in the human rights and politics theme was by far the greatest (n=5).. Labour markets, gender divides and marginalisation were amongst the common concerns raised:

*ESH: “Exploitation of workers from the developing world.”*

*ESH: “Gender gap, more pronounced than other typical technological gaps/digital divides.”*

*ESH: “Known biases about minorities and marginalised people.”*

#### Core Forum

The AI table at the knowledge café was attended by up to 16 participants at any one time. The AI discussion platforms (knowledge café, online space and boards) received a total of 49 contributions throughout the day. We identified four broad themes arising from the discussion on AI: critical perception (n= 21); current uses (n=19); training wants/needs (n= 7); and specific tools (n= 2). Detailed findings of the CORE Forum AI discussion and independent synthesis have been published elsewhere.^18^ Here, we give a brief overview of the critical perception findings.

Within the critical perception theme, we saw issues raised around trustworthiness and bias. This was underpinned by themes of AI’s rapidly evolving nature and a lack of fit-for-purpose tools, and a lack of expertise in the workforce:

*CORE: “Concern about assumption that the data ingested in LLMs is all valid and reliable. And lack of non-western /European content which continues to skew what’s generated by genAI tools.”*

*CORE: “Often find that it doesn't interpret what you're intending and required a lot of tweaking to get the outcome you want/need.”*

*CORE: “Hard to keep up - the more I know the more I realise I don’t know.”*

Despite the issues, we saw themes arising around application of AI tools and motivation to use them:

*CORE: “I use AI to explore search terms, create presentations for teaching and conferences. I'd like to use it more to speed up some more boring tasks that are part of my job.”*

*CORE: “Use Google Notebook LLM for evidence summaries using results and picking out themes from them.”*

#### SR Conversations

We received 53 contributions to the question regarding concerns. We also received a further 18 comments regarding what a new methodology should look like, however these were not taken forward for analysis in this space.

We derived four broad themes from across the 53 responses: functionality and reporting (n = 27); workforce (n = 17); ethics (n = 5); and environmental (n = 4). Within functionality and reporting, almost half of the responses related to transparency and reproducibility (n = 13):

*SRC: “Generative AI seems to undermine the transparency and reproducibility principles of SR”*

*SRC: “The fact that you can ask an LLM or genAI the same question and get a different answer each time is a big concern for systematic reviews, where the expectation is that you report your methods transparently, and somebody else should be able to replicate it (e.g. reporting search strategies in full so they could be reproduced). Many AI tools are a bit of a black box that mean this isn't possible.”*

*SRC: “Concerns of transparency of data: how the data are harvest or created.”*

A substantial portion of the workforce responses related to a perceived skills gap (n = 9, 53%), with comments around the time taken to learn how to use AI tools and the fast-paced advances in AI:

*SRC: “Time taken to learn how to use the AI - this might take longer than doing it from scratch myself!”*

*SRC: “Being required to teach AI but unable to keep up with the newest technology, completely overwhelmed.”*

A selection suggested AI may be creating a skills gap in the workforce due to lose of existing skills (n = 3):

*SRC: “I will lose skills if I become too dependent upon AI. Lose skills in verifying what AI does”*

*SRC: “We're not learning/improving ourselves anymore (to write emails, to translate...), we're using shortcuts.”*

*SRC: “Sifting and reading all the literature myself gives me a feel for, and broader general knowledge of, my research area. Using AI to reduce the time spent in the literature, whilst convenient, might make me less 'expert' as time goes on. I worry my conclusions would be weaker as my knowledge of the theoretical area decreases”*

#### HS Survey

We utilised 41 free text answers given across the survey to derive themes, resulting in five broad themes: Confidence (n = 5); Readiness levels (n = 9); Data management (n = 14); Human factors (n = 11); and Environment (n = 2).

Comments around errors and misinformation arising from the use of AI dominated the data management theme (n = 9):

*HS: “I have experienced AI giving me false information, for example, recommended papers to cite. These papers did not exist but the AI was happy to make a write up using them as key sources.”*

*HS: “I've spent a lot of time correcting AI derived data extraction.”*

*HS: “For any complex data that requires inference, I'd rather just do it myself as in my experience AI tools are inaccurate about 50% of the time.”*

Under the theme of readiness levels, many participants believed that AI should only be used in a supporting role (n = 8):

*HS: “It may be useful in screening and data extraction. However, it does always require a human to check results and may struggle in more complex study designs (e.g. diagnostics vs RCTs).”*

*HS: “I think it has many applications but more in the 'grunt work' phase, sourcing and downloading PDFs, compiling a list of results from one database to another etc. Any tasks requiring nuance and inference I think should be left to a human.”*

We specifically asked participants for the ‘top 5 concerns’ they had about using AI to support horizon scanning and received 46 responses. These were broadly categorised, with reliability/accuracy and training, skillset and guidance being identified as the main concerns (Table 2). This echoed the thematic findings where data management and human factors were prominent.

When asked to select how they felt about using AI driven recommendations or outputs, half of the participants were somewhat uncomfortable or extremely uncomfortable (n = 6). A quarter were neutral (n = 3) and the remaining where somewhat comfortable (n = 3). Interestingly, 10 participants indicated that they thought AI could be used to benefit horizon scanning, yet only three thought data quality and reliability would be positively impacted by using AI.
