# Supplementary material for: Concerns of AI use in evidence synthesis based practices: collective views from the community
Source: BMC Med Res Methodol. 2026 Mar 28;26:106. doi: 10.1186/s12874-026-02844-x (PMC13151256; doi:10.1186/s12874-026-02844-x)
Supplement: Supplementary file 2 — Supplementary Material 2. [file 12874_2026_2844_MOESM2_ESM.docx]

# Supplement 1: Qualtrics Horizon Scanning Survey

Start of Block: Welcome

Information sheet **Artificial intelligence for horizon scanning**  We appreciate your interest in participating in this questionnaire. Before you decide whether or not you wish to take part it is important that you understand why the research is being done and what it will involve. Please read this information carefully and discuss it with others if you wish. **What is the purpose of the research?**  The Innovation Observatory wants to understand what worries people about using artificial intelligence (AI) in health and social care research. We are especially focused on a type of research called horizon scanning. Many research fields have reporting standards to show their work is reliable, and recently, these standards have been expanded to include AI. We believe a reporting standard could help make AI use in horizon scanning more transparent. Your feedback will help us determine what information researchers should share in a report and how detailed it should be to ensure AI methods are clear and understandable. This will directly inform the development of a new reporting standard and help us to implement AI responsibly within the Innovation Observatory systems. **What is horizon scanning?**  Horizon scanning is a methodology used to capture signals, trends and insights. It belongs to a family of methodologies called futures and foresight analyses. The Innovation Observatory uses horizon scanning to inform our stakeholders (policy makers, decision makers, regulators, NHS groups, and commissioning groups) of which medical technologies or medicines are being trialled and for what medical conditions. This helps them to understand future changes, challenges, and priorities, and make informed decisions about the direction and economic implications of healthcare practice. **What do we mean by Artificial Intelligence?**  AI is a broad term encompassing many methodologies such as machine learning, natural language processing, large language models or generative AI, and voice recognition software. Some examples of tools using AI include: Conversational software such as ChatGPT and Gemini, systems like Alexa or Siri, or specialised coding packages like Scikit-learn and PyTorch. We are interested in any form of AI and how you might use it. For example you may run complex algorithms to refine your data, or simply use Siri to ask questions. Equally, we want to know if you don't use it at all, and why that might be. **Who can take part?** Anybody who is involved in producing horizon scans or uses the outputs from horizon scans in their own work. You do not need specialist knowledge of horizon scanning or artificial intelligence to take part in this questionnaire. **What does taking part involve?**  For this piece of research, we will ask you to answer two sets of questions. The first set will be around your involvement in horizon scanning and your use of artificial intelligence in your own work. The second set will be about using artificial intelligence in the context of horizon scanning for health and social care research. The questionnaire will take approximately 25 minutes to complete and no background knowledge is required. **Do I have to take part?**  No. Please note that participation is voluntary. If you do decide to take part, you may withdraw at any point for any reason before submitting your answers by pressing the ‘Exit’ button/ closing the browser. **What information will be collected and who will have access to the information collected?**  We will not collect any data that could directly identify you. We will take all reasonable measures to ensure that data remain confidential. The responses you provide will be stored in a password-protected electronic file on Newcastle University secure servers and may be used in academic publications, conference presentations, reports for external organisations, or workshops. We may also use direct quotes from your responses in our research outputs. If you agree to take part in the research study the data provided will be made available as “open data” through a research data repository, https://data.ncl.ac.uk/. This means the study data will be publicly available and may be used for purposes not related to this study. It will not be possible to identify you from the “open data”. **What are the possible benefits of taking part?**  There are no direct benefits of taking part in this research. The most likely benefits may be experienced by others, in the future, as a consequence of discovery through research. **What are the possible disadvantages and risks of taking part?**  There are no known disadvantages to taking part. **Who is the sponsor and data controller for this research?**  Newcastle University is the sponsor for this study based in the United Kingdom. Newcastle University will be using information from you in order to undertake this study and will act as the data controller for this study. This means that Newcastle University is responsible for looking after your information and using it properly. The lawful basis for carrying out this study under GDPR is Task in the Public Interest, (Article 6,1e) as research is cited as part of the University’s duties. The lawful basis for processing any special categories of personal data is Scientific Research (Article 9,2j). Your rights to access, change or move your information are limited, as Newcastle University need to manage your information in specific ways in order for the research to be reliable and accurate. If you withdraw from the study, Newcastle University will keep the information about you that has already been obtained. To safeguard your rights, the minimum personally-identifiable information will be used. You can find out more about how Newcastle University uses your information at https://www.ncl.ac.uk/data-protection/data-protection-policy and/or by contacting their Data Protection Officer Maureen Wilkinson, rec-man@ncl.ac.uk. **Who is funding this research?**  NIHR Innovation Observatory at Newcastle University. **Has this study received ethical approval?**  This research has been reviewed by, and received ethics clearance through, Newcastle University [57600/2023]. **Who should I contact for further information relating to the research?**  For further information regarding the study or to arrange a meeting with a member of the study team, you can contact the primary investigator who will help to arrange this: Hannah O’Keefe, hannah.o’keefe@newcastle.ac.uk **Who should I contact in order to file a complaint?**  If you wish to raise a complaint about the study you can contact the primary investigator: Hannah O’Keefe, hannah.o’keefe@newcastle.ac.uk, or programme manager: Christopher Marshall, chris.marshall@newcastle.ac.uk. Your complaint will be investigated, and appropriate action will be taken. If you wish to raise a complaint on how your personal data is handled, you can contact the Data Protection Officer who will investigate the matter: Maureen Wilkinson, rec-man@ncl.ac.uk. If you are not satisfied with their response you can complain to the Information Commissioner’s Office (ICO): https://ico.org.uk/ **Please indicate that you have read the information above and agree to participate with the understanding that the data you submit will be processed accordingly.**

- I have read the information above and consent to the terms of the research (1)

End of Block: Welcome

Start of Block: Before you begin

Descriptions **What is horizon scanning?** Horizon scanning is a methodology used to capture signals, trends and insights. It belongs to a family of methodologies called futures and foresight analyses. The Innovation Observatory uses horizon scanning to inform our stakeholders (policy makers, decision makers, regulators, NHS groups, and commissioning groups) of which medical technologies or medicines are being trialled and for what medical conditions. This helps them to understand future changes, challenges, and priorities, and make informed decisions about the direction and economic implications of healthcare practice. **What do we mean by Artificial Intelligence?**  AI is a broad term encompassing many methodologies such as machine learning, natural language processing, large language models or generative AI, and voice recognition software. Some examples of tools using AI include: Conversational software such as ChatGPT and Gemini, systems like Alexa or Siri, or specialised coding packages like Scikit-learn and PyTorch. We are interested in any form of AI and how you might use it. For example you may run complex algorithms to refine your data, or simply use Siri to ask questions. Equally, we want to know if you don't use it at all, and why that might be.

End of Block: Before you begin

Start of Block: Artificial Intelligence at work

1 How often do you come across horizon scanning in your own work?

- All the time (1)
- Sometimes (2)
- Rarely (3)
- Never (4)

2 How are you involved in horizon scanning?

- I conduct horizon scanning in my work (1)
- I use the outputs from horizon scans in my work (2)
- Other (3)
- I am not involved with horizon scans (4)

Display this question:

If 2 = 3

3 Please tell us how you are involved in horizon scanning.

________________________________________________________________

________________________________________________________________

________________________________________________________________

________________________________________________________________

________________________________________________________________

End of Block: Artificial Intelligence at work

Start of Block: Artificial Intelligence at work

4 Do you use any form of artificial intelligence in part of your daily work? For this question, we would like to know about your work in general, not just horizon scanning.

- All the time (1)
- Sometimes (2)
- Rarely (3)
- Never (4)
- Don't know (5)

Display this question:

If 4 != 4

Or 4 != 5

5 What type of artificial intelligence do you use?

- Machine learning (1)
- Natural language processing (2)
- Large language models (3)
- Generative AI (4)
- Voice recognition (5)
- Other (6)
- Don't know (7)

Display this question:

If 4 != 4

Or 4 != 5

6 Please tell us what you use artificial intelligence for. Do you use any specific named tools?

________________________________________________________________

________________________________________________________________

________________________________________________________________

________________________________________________________________

________________________________________________________________

End of Block: Artificial Intelligence at work

Start of Block: Artificial Intelligence at work

7 In general how confident are you at using artificial intelligence methods for small tasks? By small tasks we mean things like document editing, generating images or visuals, transcribing or summarising meeting recordings.

- Extremely confident (1)
- Somewhat confident (2)
- Neither confident or unconfident (3)
- Somewhat unconfident (4)
- Not confident at all (5)

8 In general how confident are you at using artificial intelligence methods for advanced tasks? By advanced tasks we mean things like classification systems, data handling and extraction, and building your own models.

- Extremely confident (1)
- Somewhat confident (2)
- Neither confident or unconfident (3)
- Somewhat unconfident (4)
- Not confident at all (5)

9 Thinking about these two questions, can you explain why you feel this way?

________________________________________________________________

________________________________________________________________

________________________________________________________________

________________________________________________________________

________________________________________________________________

End of Block: Artificial Intelligence at work

Start of Block: Artificial Intelligence for Horizon Scanning

10 The next few questions relate specifically to horizon scanning for health and social care research. Please answer as fully and honestly as you can.

11 Do you think artificial intelligence should be used to support horizon scanning?

- Yes (1)
- No (2)
- Don't know (3)

12 Can you expand on why you think this?

________________________________________________________________

________________________________________________________________

________________________________________________________________

________________________________________________________________

________________________________________________________________

End of Block: Artificial Intelligence for Horizon Scanning

Start of Block: Artificial Intelligence for Horizon Scanning

13 If artificial intelligence was to be used, which parts of the horizon scanning process do you think would benefit most and why?

________________________________________________________________

________________________________________________________________

________________________________________________________________

________________________________________________________________

________________________________________________________________

End of Block: Artificial Intelligence for Horizon Scanning

Start of Block: Artificial Intelligence for Horizon Scanning

14 If artificial intelligence was to be used, which parts if the horizon scanning process do you think are least likely to benefit and why?

________________________________________________________________

________________________________________________________________

________________________________________________________________

________________________________________________________________

________________________________________________________________

End of Block: Artificial Intelligence for Horizon Scanning

Start of Block: Artificial Intelligence for Horizon Scanning

15 Would you feel comfortable relying on artificial intelligence driven recommendations or outputs?

- Yes (1)
- No (2)
- Don't know (3)

16 In general how comfortable/uncomfortable would you feel using artificial intelligence driven recommendations or outputs?

- Extremely uncomfortable (1)
- Somewhat uncomfortable (2)
- Neither comfortable nor uncomfortable (3)
- Somewhat comfortable (4)
- Extremely comfortable (5)

17 Thinking of these two question, can you expand on why you feel this way?

________________________________________________________________

________________________________________________________________

________________________________________________________________

________________________________________________________________

________________________________________________________________

End of Block: Artificial Intelligence for Horizon Scanning

Start of Block: Artificial Intelligence for Horizon Scanning

18 Do you think data quality and reliability are positively or negatively impacted by using artificial intelligence methods?

- Positively impacted (1)
- Neither positively or negatively impacted (2)
- Negatively impacted (3)
- Don't know (4)

19 Can you expand on why you think this?

________________________________________________________________

________________________________________________________________

________________________________________________________________

________________________________________________________________

________________________________________________________________

20 Are there specific factors associated with artificial intelligence that you think could positively or negatively affect data quality and reliability?

________________________________________________________________

________________________________________________________________

________________________________________________________________

________________________________________________________________

________________________________________________________________

End of Block: Artificial Intelligence for Horizon Scanning

Start of Block: Artificial Intelligence for Horizon Scanning

21 Thinking about conversations you may have had about using artificial intelligence in a horizon scanning context, have other people mentioned things that worry or concern them?

- Yes (1)
- No (2)
- Don't know (3)

Display this question:

If 21 = 1

22 Can you expand on what concerns or worries people have mentioned? Please be as detailed as possible

________________________________________________________________

________________________________________________________________

________________________________________________________________

________________________________________________________________

________________________________________________________________

End of Block: Artificial Intelligence for Horizon Scanning

Start of Block: Artificial Intelligence for Horizon Scanning

23 What are the top 5 things that worry or concern you about using artificial intelligence to support horizon scanning? It may be useful to think about:      Implementation factors - such as transparency, accuracy, bias, explainability, ethics, cost      Personal factors - such as knowledge, understanding, training needs, time commitments      Global factors - such as environment, workforce, labour, economics

- 1. (1) __________________________________________________
- 2. (2) __________________________________________________
- 3. (3) __________________________________________________
- 4. (4) __________________________________________________
- 5. (5) __________________________________________________

End of Block: Artificial Intelligence for Horizon Scanning

Start of Block: Block 12

24 Do you have any other comments you would like to make?

________________________________________________________________

________________________________________________________________

________________________________________________________________

________________________________________________________________

________________________________________________________________

End of Block: Block 12
